# No Evidence for Cross-Modal fMRI Adaptation in Macaque Parieto-Premotor Mirror Neuron Regions

**DOI:** 10.3390/brainsci13101466

**Published:** 2023-10-17

**Authors:** Saloni Sharma, Koen Nelissen

**Affiliations:** 1 Laboratory for Neuro- & Psychophysiology, Department of Neurosciences, KU Leuven, 3000 Leuven, Belgium; saloni_sharma@hms.harvard.edu; 2 Leuven Brain Institute, KU Leuven, 3000 Leuven, Belgium

**Keywords:** macaque, fMRI, adaptation, cross-modal, action, motor

## Abstract

To probe the presence of mirror neurons in the human brain, cross-modal fMRI adaptation has been suggested as a suitable technique. The rationale behind this suggestion is that this technique allows making more accurate inferences about neural response properties underlying fMRI voxel activations, beyond merely showing shared voxels that are active during both action observation and execution. However, the validity of using cross-modal fMRI adaptation to demonstrate the presence of mirror neurons in parietal and premotor brain regions has been questioned given the inconsistent and weak results obtained in human studies. A better understanding of cross-modal fMRI adaptation effects in the macaque brain is required as the rationale for using this approach is based on several assumptions related to macaque mirror neuron response properties that still need validation. Here, we conducted a cross-modal fMRI adaptation study in macaque monkeys, using the same action execution and action observation tasks that successfully yielded mirror neuron region cross-modal action decoding in a previous monkey MVPA study. We scanned two male rhesus monkeys while they first executed a sequence of either reach-and-grasp or reach-and-touch hand actions and then observed a video of a human actor performing these motor acts. Both whole-brain and region-of-interest analyses failed to demonstrate cross-modal fMRI adaptation effects in parietal and premotor mirror neuron regions. Our results, in line with previous findings in non-human primates, show that cross-modal motor-to-visual fMRI adaptation is not easily detected in monkey brain regions known to house mirror neurons. Thus, our results advocate caution in using cross-modal fMRI adaptation as a method to infer whether mirror neurons can be found in the primate brain.

## 1. Introduction

In the field of neuroimaging, understanding neural responses at a fine-grained level has been a challenge due to the poor spatial resolution of typical fMRI experiments. For instance, overlapping fMRI responses in certain brain regions during two distinct task conditions could be driven by two independent subpopulations of neurons within these activated voxels or it could reflect activity from a single population of neurons responding to both conditions. Despite this shortcoming, two techniques have emerged to overcome the limitations of the voxel resolution and enable the inference of neural population response properties—fMRI adaptation (repetition suppression) and multivariate patterns analysis (MVPA).

One area of research where employing fMRI to infer neural response properties becomes particularly relevant is when examining the occurrence of mirror neurons in the primate brain [1,2]. The premise of using fMRI adaptation to discern the presence of (multimodal) mirror neurons is based upon the assumption that regions containing mirror neurons should exhibit adaptation (reduced fMRI responses) when a specific motor act is repeated, whether it is executed or observed. However, the few studies employing fMRI adaptation to probe the existence of mirror neurons in the human brain produced discrepant results, both with respect to the nature of observed adaptation effects (uni- and/or cross-modal) and the specific brain regions where these effects are observed [3,4,5,6,7,8]. Moreover, most studies did not find evidence for fMRI adaptation in presumed homologs of monkey parietal and premotor mirror neuron regions in the human brain. 

Besides the ongoing debate on whether repetition suppression can accurately infer neural selectivity and the precise nature of the neuronal mechanisms underlying repetition suppression [9,10,11,12,13,14], there are currently limited data regarding possible uni-modal or cross-modal adaptation effects at the single-cell or population level in monkey mirror neuron regions [15,16]. Previous work from our lab has shown uni-modal visual fMRI adaptation effects in early visual cortices but did not find uni- or cross-modal fMRI adaptation effects in monkey parietal and premotor mirror neuron regions [2]. Given that cross-modal fMRI adaptation effects depend on numerous factors, including task conditions and presentation design [9], here we conducted a new study using a different fMRI adaptation design, involving the same motor tasks and action videos that successfully yielded cross-modal action decoding in a previous monkey MVPA study [1]. Since uni-modal visual or motor adaptation effects may not necessarily indicate the presence of visuo-motor neurons in these voxels, and because visual-to-motor adaptation effects could potentially be attributed to priming effects, motor-to-visual adaptation has been suggested as the most likely approach for inferring the presence of mirror neurons in the underlying voxels [3,5,8]. Thus, in our current study, we specifically examined the presence of cross-modal motor-to-visual fMRI adaptation effects in the monkey brain. 

Two male rhesus monkeys were trained to perform a series of reach-and-grasp or reach-and-touch actions without visual feedback, and then to observe a video depicting a human actor executing a similar reach-and-grasp or reach-and-touch action. If monkey parietal and premotor mirror neuron regions were to show cross-modal fMRI adaptation, then we should observe a reduction in response during the observation of the same action that the monkeys had previously executed. However, neither whole-brain nor region-of-interest analyses revealed a reduction in response in monkey parietal and premotor mirror neuron regions. This finding could not be explained by the inability of the motor/visual actions to elicit responses since two independent localizers (action execution-only and action observation-only), using the same motor and visual tasks as used in the current adaptation experiment, did yield significant responses in the parietal and premotor sectors of the mirror neuron system. Furthermore, in a previous study [1], we have shown that cross-modal action decoding in monkey mirror neuron regions, using the same motor and visual tasks as employed in the current study, could be successfully demonstrated using multi-voxel pattern analysis. Overall, our results suggest that mirror neuron activity does not readily translate into detectable cross-modal adaptation using fMRI. Taken together, our current and previous observations [2], along with the lack of consistent evidence for cross-modal fMRI adaptation in previous human studies, suggest that caution is needed when using cross-modal fMRI adaptation to draw inferences about the occurrence of mirror neurons in the primate brain. 

## 2. Methods

### 2.1. Subjects

Two male monkeys (Macaca mulatta, 8–9 years old, 6–7 kg) participated in the task-based fMRI experiments to localize brain regions responding to action execution and observation and consequently in the cross-modal fMRI adaptation experiment. Animal care and experimental procedures were approved by the animal ethical committee of KU Leuven and followed national and European guidelines.

### 2.2. Fixation Training

The monkey subjects faced a liquid crystal display (LCD) screen and sat in a sphinx position in a plastic monkey chair that was placed in a mock scanner. The monkeys were trained to maintain fixation within a 2 × 2° window centered on a red dot (0.35 × 0.35°) in the center of the screen. Eye position was monitored at 120 Hz using pupil tracking and corneal reflection (Iscan Inc., Woburn, MA, USA). As long as the monkeys fixated on the red dot, they were rewarded with fruit juice. When the monkeys could successfully fixate for at least 85% of the run duration, they were consequently trained to perform motor tasks.

### 2.3. Action Execution Localizer

**Figure 1:**
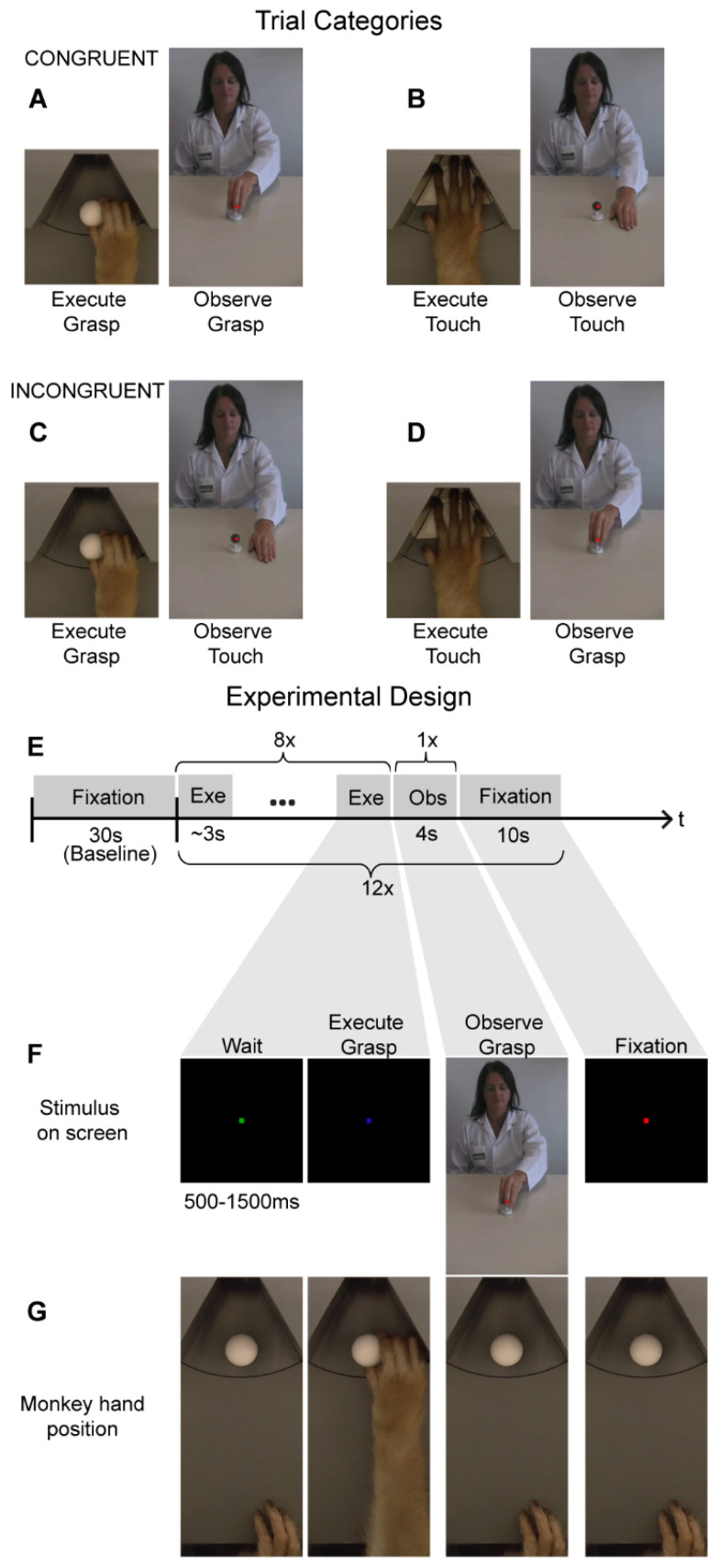
Cross--modal fMRI adaptation stimuli and experimental design: (**A**–**D**). The monkeys were tested on four different cross-modal motor-to-visual adaptation trials. Congruent trials consisted of either reach-and-grasp execution followed by reach-and-grasp observation (**A**) or reach-and-touch execution followed by reach-and-touch observation (**B**). Incongruent trials consisted of reach-and-grasp execution followed by reach-and-touch observation (**C**), or reach-and-touch execution followed by reach-and-grasp observation (**D**). (**E**) A cross-modal fMRI adaptation fMRI run consisted of thirty seconds of fixation-only (which served as baseline) followed by 12 adaptation trials. An adaptation trial consisted of a sequence of eight action executions, followed by observation of one action video and ten seconds of fixation-only. (**F**,**G**) An example of a congruent reach-and-grasp adaptation trial depicting the visual stimuli displayed on the MR-compatible screen in front of the monkey (**F**) and the corresponding hand position of the monkey (**G**).

The monkeys were trained to perform two different motor tasks in the dark: reach-and-grasp (Figure 1A) or reach-and-touch (Figure 1B). The motor task follows similar procedures as previously described [1,17,18,19] and will be described here only briefly. A reach-and-grasp trial started when the monkey placed its right hand in the start position and fixated on a green fixation point displayed centrally on the screen before him. Breaking of fixation or movement of the hand resulted in the trial being aborted and a yellow cross appeared until the monkey either placed his hand in the start position or started fixating again. After a fixed time of 5000 ms during which the monkeys received one juice award at a randomized time in every trial, the green fixation point changed to blue, indicating to the monkey that he could now reach and grasp the object, positioned on an MR-compatible turntable in front of him, with the right hand. 

If the monkey did not reach and grasp/lift the object within 2000 ms after the cue turned blue, this would result in the trial being aborted. Once the object (a sphere of 23 mm radius) had been grasped, the monkey was required to lift it 5 mm and maintain that position for at least 500 ms to receive a juice reward. Once the monkey was rewarded for a successful trial, a new trial only started (green fixation point) once the monkey’s hand was back to the initial start position while maintaining fixation. Optic fiber cables were located at the start and end positions of the action execution trajectory, as well as at two different locations along this hand/arm trajectory to be able to track the location of the monkeys’ hand/arm and to record the timing of the execution of the motor tasks. The second motor task consisted of a reach-and-touch action that did not involve grasping an object. For this motor task, the MR-compatible turntable was positioned so that an empty slot (not containing an object) was positioned in front of the monkey. The monkey subject was required to place its hand onto this empty slot on the disk. After reaching forward, the monkey was required to leave its open hand (palm facing down) on the disk for at least 500 ms to receive the juice reward. The visual stimuli used to cue the monkey and the timing parameters were the same as during the reach-and-grasp task. Both motor tasks were performed in the dark to avoid neural modulations due to visual feedback from the hand and arm or the object [1]. During the fMRI adaptation experiments, monkeys performed the same motor tasks.

### 2.4. Action Observation Localizer

In this visual localizer experiment, monkeys observed blocks of videos depicting a human actor performing either a reach-and-grasp (Figure 1A) or a reach-and-touch action (Figure 1B). The size of the videos was 8.5 × 14 visual degrees. The duration of the videos was 4 s. During these visual blocks, monkeys were rewarded for fixating on a small red dot displayed in the center of the screen and for maintaining the right hand in the start position while the videos were presented. Along with the action observation blocks, each run also contained fixation-only blocks (which served as baseline), during which monkeys were rewarded for fixating on a red fixation point only and keeping the right hand in the start position. In a case where the monkey broke fixation or removed its hand from the start position at any point during a run, reward delivery was immediately stopped. Hand and eye position were monitored using optic fibers and an MR compatible camera, respectively (Iscan Inc.). During the fMRI adaptation experiments, monkeys observed the same action videos as used in this visual localizer.

### 2.5. Cross-Modal fMRI Adaptation

For the fMRI adaptation experiment (Figure 1), we specifically examined cross-modal motor-to-visual adaptation by comparing MR responses during action observation in congruent (Figure 1A,B) vs. incongruent (Figure 1C,D) trials. Congruent trials consisted of a sequence of executions of a particular motor action (either reach-and-grasp or reach-and-touch), followed by a video depicting a human actor performing an action with the same goal (reach-and-grasp or reach-and-touch, respectively). The size of the videos was 8.5 × 14 visual degrees. We opted to use these videos since previous work from our lab has demonstrated their effectiveness in driving mirror neuron regions using both univariate fMRI analysis and MVPA [1]. Thus, any possible observed absence of fMRI adaptation effects in the current study could not merely be attributed to the ineffectiveness of the visual stimuli in activating macaque parieto-frontal mirror neuron regions.

A typical fMRI adaptation run started with 30 s of fixation-only where the monkeys simply observed a red dot in the center of the screen while maintaining the right hand in the start position (Figure 1E). This block of fixation was followed by twelve cross-modal adaptation trials, each of which consisted of a block of eight reach-and-grasp or reach-and-touch motor executions, followed by presentation of one action video (4 s duration) and a ten-second fixation-only period (Figure 1E). Here, the motor tasks were similar to the action execution localizer where a motor trial started when the monkey fixated on a green cue on the screen in front of him (Figure 1F). After a random time of 500–1500 ms, the green cue turned blue indicating that the monkey should either reach forward to grasp and lift an object (sphere of 23 mm radius; Figure 1F,G) or simply place his hand (palm facing down) on an empty slot of the MR-compatible disk. Note that the reach-and-grasp and reach-and-touch execution tasks were performed in complete darkness with the same visual cues for both tasks. Thus, the monkey only knew which of the two motor actions had to be accomplished by the presence or absence of an object after reaching forward. Nevertheless, once the monkey had reached forward, he had to lift and grasp the object or place his palm down, depending on the condition, for a full 500 ms for the trial to be considered successful and for the monkey to receive juice reward. Further, only after the monkey had correctly executed eight of these actions, was a video presented (four-second duration) depicting a human actor performing a similar reach-and-grasp or a reach-and-touch action (Figure 1E,F). The video was not displayed until the monkey’s hand was back in the start position to ensure there was no hand movement during action observation. During the presentation of this video, the monkey was required to maintain fixation on a red dot in the center of the screen within a 2 × 2° fixation window and keep his hand at the start position. Further, we centered the video such that the motor action occurred foveally where the monkey was fixating to ensure that the action was observed. After 4 s, the video would disappear and only the red dot remained for ten seconds before a new adaptation trial (consisting of eight motor actions followed by observing one action video and a 10 s fixation-only period) began. The macaque motor actions and videos depicting the human actions used here are the same as those used in the action execution and observation localizers (see previously). Hand and eye position were monitored using optic fibers and an MR-compatible camera, respectively (Iscan Inc.).

### 2.6. fMRI Data Acquisition

Data were acquired in a Siemens 3T full body scanner (Prisma^fit^) using a gradient-echo T2*-weighted echo-planar imaging sequence of 40 horizontal slices (action observation localizer; TR = 2 s, TE = 18 ms, flip angle = 90°, 1.25 mm isotropic) or 42 horizontal slices (action execution localizer and cross-modal fMRI adaptation experiments; TR = 2 s, TE = 17 ms, flip angle = 90°, 1.25 × 1.25 × 1.2 mm). An in-house designed and manufactured eight-channel phased array coil and a saddle-shaped, radial transmit-only surface coil were used for data acquisition. We intravenously injected an iron contrast agent (Molday ION, BioPAL; 8–12 mg/kg) before each scanning session, an iron contrast agent to increase signal-to-noise ratio [20].

### 2.7. Experimental Design

For the action execution localizer, a block design was used consisting of three conditions: execute reach-and-grasp, execute reach-and-touch, and fixation-only (baseline). A typical run consisted of 5 start volumes and was followed by a sequence of blocks of 20 volumes or 40 s reach-and-grasp sphere 23 mm—10 volumes or 20 s fix to 40 s reach-and-touch—20 s fix to 40 s fix baseline—20 s fix, repeated twice in the same run. A full run hence consisted of 185 volumes. A different presentation order for these three conditions was used in each run. In a full run, the monkeys could perform ten reach-and-grasp or reach-and-touch motor actions each, and only runs where they had executed eight or more motor actions were used for single-subject analysis. Thus, for both monkeys M1 and M2, 15 runs each were used for data analysis. 

For the action observation localizer, a block design was used that contained three conditions: observe reach-and-grasp, observe reach-and-touch, and fixation-only baseline. Each block (condition) lasted for 10 volumes (20 s) and was presented six times in one run. Each run consisted of 185 volumes (5 start volumes, followed by 6 repeats of the 3 conditions). In total, six different orders of presentation were used for the fMRI runs. For single-subject data analysis, only runs where the monkeys fixated for 90% or more of the run duration were used, resulting in 20 runs each from monkey M1 and monkey M2. 

**Figure 2:**
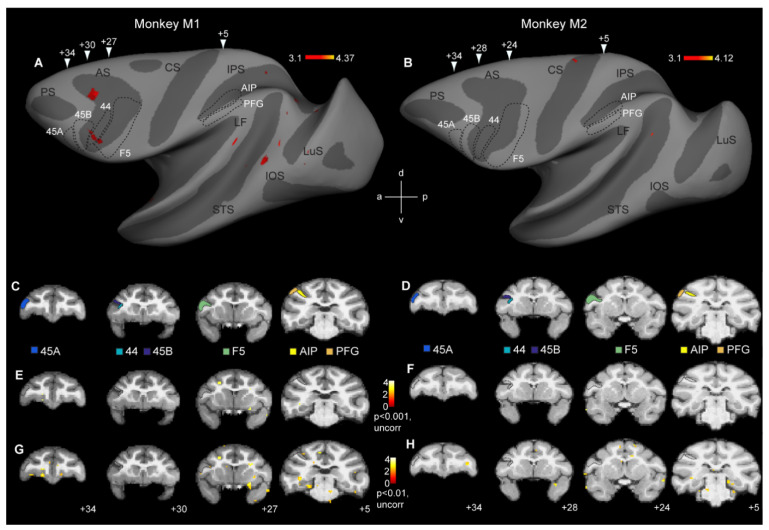
Whole-brain analysis fails to reveal cross-modal fMRI adaptation responses in monkey mirror neuron regions: (**A**,**B**) Contrasting incongruent to congruent conditions at *p* < 0.001 (uncorrected) only yielded sparse effects in monkey M1 (**A**), while monkey M2 failed to show any repetition suppression effects (**B**). Arrowheads indicate approximate locations of the coronal sections shown in (**C**,**D**). (**C**,**D**) Coronal sections showing the locations of six regions of interest in monkey M1 (**C**) and monkey M2 (**D**): parietal areas AIP and PFG, premotor area F5, area 44, and prefrontal areas 45B and 45A. (**E**,**F**) Same contrast as shown in (**A**,**B**), depicting cross-modal repetition suppression effects in monkey M1 (**E**) and monkey M2 (**F**) at *p* < 0.001, uncorrected. (**G**,**H**) Coronal sections showing the contrast *incongruent* vs. *congruent* in monkey M1 (**G**) and monkey M2 (**H**) at *p* < 0.01, uncorrected. Black stippled lines, both on the inflated brain and coronal sections indicate the approximate locations of the frontal and parietal regions of interest. LuS = lunate sulcus, IOS = inferior occipital sulcus, STS = superior temporal sulcus, LF = lateral fissure, IPS = intraparietal sulcus, CS = central sulcus, AS = arcuate sulcus, d = dorsal, v = ventral, a = anterior, p = posterior.

**Figure 3:**
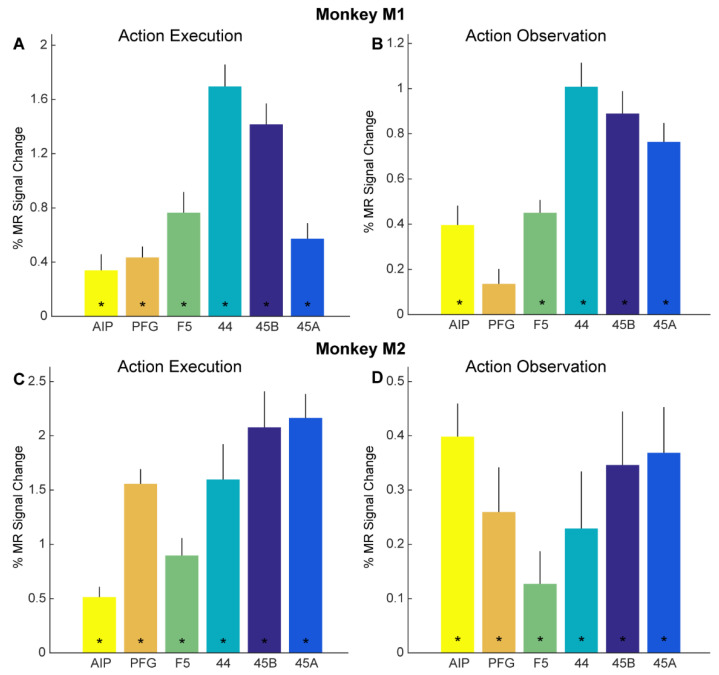
Functional MRI responses for action execution and observation localizers: (**A**,**B**) Bar plots showing percent MR signal change for planning/executing (**A**) and observing (**B**) reach-and-grasp and reach-and-touch actions (compared to fixation-only baseline) for anatomically defined parietal ROIs AIP (yellow) and PFG (orange), premotor F5 (green), prefrontal 44 (turquoise), 45B (dark blue) and 45A (light blue) in monkey M1. Asterisks indicate significance at *p* < 0.05, Bonferroni corrected for number of ROIs. Error bars indicate standard error of the mean across runs. (**C**,**D**). Bar plots show percent MR signal change for executing (**C**) and observing (**D**) reach-and-grasp and reach-and-touch actions (compared to fixation-only baseline) for anatomically defined ROIs in monkey M2. Same conventions as in (**A**,**B**).

**Figure 4:**
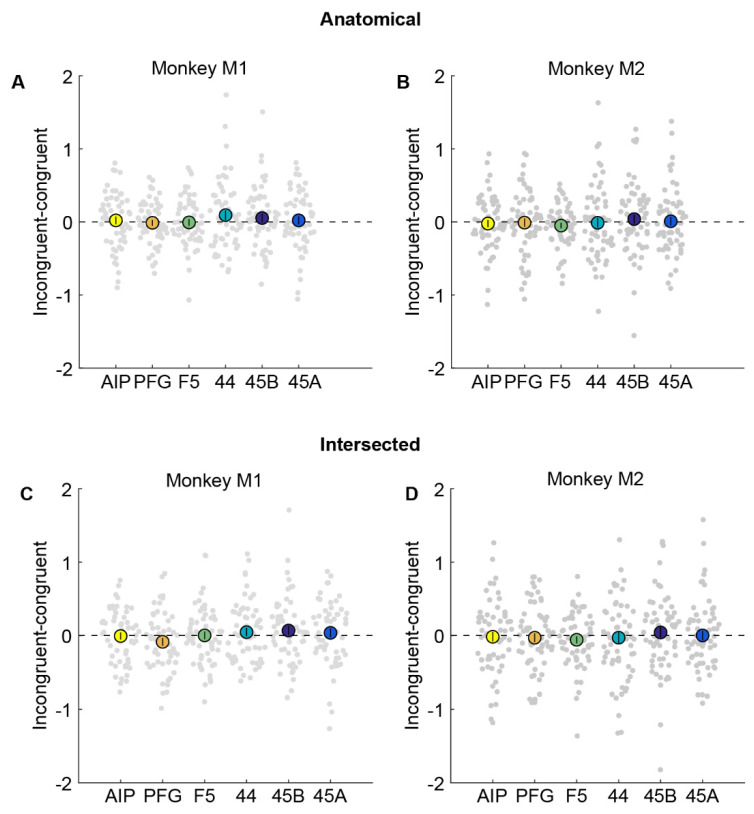
No evidence for cross-modal fMRI adaptation in anatomical or intersected ROIs. (**A**,**B**). Percentage signal change obtained by subtracting congruent from incongruent condition in anatomically defined parietal areas AIP (yellow), PFG (orange), premotor F5 (green), area 44 (turquoise), and prefrontal areas 45B (dark blue) and 45A (light blue) in monkey M1 (**A**) and monkey M2 (**B**). Filled bigger circles indicate the mean difference averaged across runs, while each small circle in the swarm chart depicts the difference per run. Error bars indicate standard error of the mean across runs. (**C**,**D**). Percentage signal change for incongruent–congruent condition in intersected parietal areas AIP, PFG, premotor F5, area 44, and prefrontal areas 45B and 45A in monkey M1 (**C**) and monkey M2 (**D**). Same conventions as in (**A**,**B**). Neither monkey showed a significant difference between congruent and incongruent trials.

For the cross-modal fMRI adaptation experiment, a run consisted of 300 volumes or 10 min in total. Each run started with a fixation-only condition for 30 s, which served as baseline. As mentioned above, a cross-modal adaptation trial consisted of a sequence of eight action execution events, followed by observing one action video (4 s duration) and followed by ten seconds of fixation-only (Figure 1E). In total, four different adaptation trials were used (Figure 1A–D): (1) Reach-and-grasp execution followed by reach-and-grasp observation (Figure 1A), (2) Reach-and-touch execution followed by reach-and-touch observation (Figure 1B), (3) Reach-and-grasp execution followed by reach-and-touch observation (Figure 1C), and (4) Reach-and-touch execution followed by reach-and-grasp observation (Figure 1D). The first two conditions were labeled as “congruent” since observation of an action (goal) was preceded by execution of a similar action (goal). In contrast, in the latter two ‘incongruent’ conditions, observation of an action (goal) was preceded by execution of a different action (goal). Each of these four trial types was repeated three times in a run (bringing it to a total of twelve adaptation trials in one run) and the order of these conditions was randomized both within and across runs. Only runs where monkeys successfully completed all twelve adaptation trials were used for further analysis. Thus, we used 56 runs from monkey M1 and 60 runs from monkey M2 in single-subject analysis. Note that these runs were collected across 4 days for monkey M1 and across 3 days for monkey M2; thus, the monkeys only completed 15–20 runs per day.

### 2.8. Data Preprocessing and GLM Fitting

Data were preprocessed using statistical parametric mapping (SPM12) software (https://www.fil.ion.ucl.ac.uk/spm/software/spm12/) and JIP (http://www.nitrc.org/projects/jip/). Spatial pre-processing consisted of realignment and non-rigid co-registration (using JIP) of the functional images to a high-resolution anatomical template of the monkey’s own brain. Functional volumes were then resliced to 1 mm^3^ isotropic and smoothed with a 1.5 mm (FWHM) Gaussian kernel. A general linear model (GLM) was then used to compute the response amplitude at each voxel using SPM12 following previously detailed procedures to fit a GLM [20,21]. To do this, a MION hemodynamic response function was convolved with a boxcar model representing the various stimulus conditions [20]. For the action execution and observation localizers, the three conditions (reach-and-grasp execution or observation, reach-and-touch execution, or observation and fixation baseline) were modeled as regressors of interest. For the cross-modal fMRI adaptation experiment, each action execution (eight reach-and-grasps or eight reach-and-touches), the four action observation conditions (grasp observation following grasp execution, grasp observation following touch execution, touch observation following touch execution, touch observation following grasp execution), and fixation baseline were modeled, resulting in twenty-one regressors. Additionally, six regressors corresponding to three rotations and translations along the *x*, *y*, and *z*-axis along with three regressors corresponding to the horizontal and vertical components of the eye movement traces and pupil diameter were included as regressors of no interest to account for head- and eye-movement-related confounds in all three experiments. For each run, a GLM was fitted for each voxel resulting in a map (beta map) for each condition of interest and the nine regressors of no interest. Here, single-subject data are presented for which functional images are warped onto the monkey’s own anatomy. For display purposes, whole-brain SPM T-maps are rendered on an inflated brain template of each monkey using FreeSurfer toolbox (FreeSurfer v6.0.0). In addition, single-subject SPM T-maps are represented on coronal sections of each monkey’s individual anatomical template. Since no cross-modal fMRI adaptation effects were found at the whole-brain level at *p* < 0.05, FWE corrected, and to avoid possible false negative results due to this stringent threshold, we explored if these adaptation effects might be observed at more liberal thresholds (*p* < 0.001, uncorrected or even *p* < 0.01, uncorrected).

### 2.9. Univariate Region-of-Interest-Based Analysis

For the univariate regions-of-interest (ROI) analyses, the average percentage signal change of all the voxels in the ROIs was calculated for each condition compared to the fixation baseline using MarsBaR (MarsBaR region-of-interest toolbox for SPM, v0.41.1). Statistical significance was tested using two-tailed t-tests with the significance threshold set at *p* < 0.05. ROIs were defined in each monkey’s own anatomical space based on previously known anatomical landmarks described in detail below. The ROIs were defined only in the left hemisphere since the monkeys performed the action execution tasks with their right hand only and, thus, the corresponding motor activations were biased to the left (contralateral) hemisphere [1,19]. For the adaptation experiment, in addition to null hypothesis testing using t-tests, a JZS Bayes factor with the default √2/2 scale parameter was estimated to provide evidence in favor of the null (or alternate) hypothesis [22].

*Anatomical ROIs:* For the ROI analysis, we focused on the three key regions of the macaque grasping mirror neuron network, In the parietal cortex, a ROI corresponding to area AIP (113 voxels in monkey M1, Figure 2C, yellow, and 102 voxels in monkey M2, Figure 2D, yellow) was defined on the anterior part of the lateral bank of the intraparietal sulcus based on previous single-cell and fMRI studies [23,24]. Additionally, a ROI corresponding to monkey area PFG (108 voxels in monkey M1, Figure 2C, orange, and 90 voxels in monkey M2, Figure 2D, orange) was delineated on the inferior parietal lobule [19,25,26]. In the ventral premotor cortex, we defined a ROI corresponding to premotor F5 (409 voxels in monkey M1, Figure 2C, green, and 415 voxels in monkey M2, Figure 2D, green). This F5 ROI included all three subdivisions of F5–F5p and F5a at different anterior–posterior positions in the posterior bank of the inferior arcuate sulcus and F5c on the adjacent cortical convexity [27,28,29]. Single-cell investigations have demonstrated mirror neurons in each of these ROIs [26,30,31,32,33,34]. Additionally, each of these regions has been previously shown to yield cross-modal action-specific representations for the same observed and executed reach-and-grasp or reach-and-touch actions [1] used in the current study.

In addition to ROIs for parietal and premotor mirror neuron regions, we also defined three ROIs in the neighboring prefrontal cortex. We delineated a ROI corresponding to dysgranular area 44 (49 voxels in monkey M1, Figure 2C, turquoise, and 50 voxels in monkey M2, Figure 2D, turquoise) in the fundus of the inferior arcuate sulcus [35,36]. Finally, in the prefrontal cortex, we also delineated two ROIs corresponding to area 45B (107 voxels in monkey M1, Figure 2C, dark blue, and 98 voxels in monkey M2, Figure 2D, dark blue) in the rostroventral bank of the arcuate sulcus and area 45A (79 voxels in monkey M1, Figure 2C, light blue, and 63 voxels in monkey M2, Figure 2D, light blue) on the convexity rostral to the arcuate sulcus [37,38,39,40]. We included these prefrontal regions in our ROI analyses because a human fMRI adaptation study suggested cross-modal fMRI adaptation in/near human BA 44/45 [4].

*Intersected ROIs:* In addition to examining cross-modal fMRI adaptation in anatomically defined ROIs, we also examined responses in the corresponding functionally defined ROIs by making an intersection of each anatomical ROI and the functional responses obtained in the two independent localizers. The rationale for this was that if cross-modal adaptation effects were weak, these effects could be missed when considering anatomically defined ROIs. Based on the localizer data, we created intersected functional ROIs by selecting those voxels from the anatomical ROIs that yielded either motor responses (at *p* < 0.01, uncorrected) during action execution (reach-and-grasp and reach-and-touch) vs. fixation-only and visual responses (at *p* < 0.01, uncorrected) during action observation (reach-and-grasp and reach-and-touch) vs. fixation-only. The numbers of voxels contained in the anatomical or functional defined ROIs in both subjects are shown in Table 1. 

## 3. Results

### 3.1. Whole-Brain Investigation of Cross-Modal fMRI Adaptation

The aim of this study was to investigate if cross-modal motor-to-visual fMRI adaptation effects could be observed in the monkey brain, particularly in parieto-premotor regions previously shown to house mirror neurons. A first analysis at the whole-brain level yielded no significant cross-modal motor-to-visual fMRI adaptation effects at *p* < 0.05 (FWE corrected). We therefore explored if these general weak effects might become clearer at more liberal thresholds. At *p* < 0.001 (uncorrected), some scattered effects were observed throughout the extrastriate and frontal cortex in the left hemisphere of M1 (Figure 2A), while in monkey M2, virtually no fMRI adaptation-related effects were observed (Figure 2B). Six regions of interest were examined in more detail (see below). These included parietal and premotor mirror neuron regions AIP, PFG, and F5, in addition to areas 44, 45B, and 45A, anterior to F5 (black stippled lines in Figure 2A,B). The location of these ROIs is shown on coronal anatomical sections in each subject (Figure 2C: monkey M1; Figure 2D: monkey M2). As can be seen in the inflated hemispheres (Figure 2A,B) or on the coronal sections (Figure 2E,F), neither of the regions containing parieto-premotor mirror neurons revealed any evidence for cross-modal adaptation, except for a discrete portion of the anterior part of left hemisphere area 44 in monkey M1 (Figure 2A). Even at a very liberal statistical threshold of *p* < 0.01 (uncorrected), whole-brain analysis did not show evidence of cross-modal adaptation effects in premotor or parietal mirror neuron regions, or in areas 44/45 (Figure 2G,H).

### 3.2. Action Execution and Action Observation fMRI Responses in Regions of Interest

Besides whole-brain analysis, we also examined in detail if adaptation effects might be revealed through a more sensitive ROI analysis. As mentioned above, we focused our ROI analysis on brain regions in the left hemisphere previously shown to house mirror neurons: parietal areas AIP and PFG, as well as ventral premotor area F5 (Figure 2C,D). In addition, we examined responses in several prefrontal regions neighboring F5, since some human fMRI adaptation studies suggested cross-modal motor-to-visual repetition suppression effects in these regions [4,7]: areas 44, 45B, and 45A (Figure 2C,D). First, we examined if these anatomically defined ROIs showed overall responses to our action execution and action observation localizers (see Methods). Planning and executing reach-and-grasp and reach-and-touch actions (vs. fixation-only baseline) yielded significant responses in all six ROIs in both monkey M1 (Figure 3A) and monkey M2 (Figure 3C). For both monkeys, significant planning/execution-related responses were observed in parietal areas AIP (monkey M1: t = 2.87, *p* = 0.012; monkey M2: t = 5.39, *p* = 9.53 × 10^−5^) and PFG (monkey M1: t = 5.34, *p* = 1.05 × 10^−4^; monkey M2: t = 11.56, *p* = 1.51 × 10^−8^), premotor F5 (monkey M1: t = 5.03, *p* = 1.84 × 10^−4^; monkey M2: t = 5.57, *p* = 6.91 × 10^−5^), area 44 in the fundus of the arcuate sulcus (monkey M1: t = 10.47, *p* = 5.25 × 10^−8^ monkey M2: t = 4.91, 2.29 × 10^−4^), area 45B (monkey M1: t = 9.19, *p* = 2.6 × 10^−7^; monkey M2: t = 6.25, *p* = 2.12 × 10^−5^), and 45A (monkey M1: t = 5.03, *p* = 1.85 × 10^−4^; monkey M2: t = 9.8, *p* = 1.19 × 10^−7^).

Figure 3B, D show the percentage signal change as bar plots of the same anatomical ROIs in both subjects (Figure 3B, M1, Figure 3D, M2) for observing the reach-and-grasp and reach-and-touch actions (compared to fixation-only baseline) from the action observation localizer. Significant visual responses for observing action compared to fixation baseline were observed in parietal area AIP for both monkeys (monkey M1: t = 4.61, *p* = 1.92 × 10^−4^; monkey M2: t = 6.55, *p* = 42.87 × 10^−6^), premotor F5 (monkey M1: t = 7.99, *p* = 1.71 × 10^−7^; monkey M2: t = 2.13, *p* = 0.046), area 44 (monkey M1: t = 9.51, *p* = 1.18 × 10^−8^; monkey M2: t = 2.17, *p* = 0.043), area 45B (monkey M1: t = 9.01, *p* = 2.75 × 10^−8^; monkey M2: t = 3.51, *p* = 0.0024), and area 45A (monkey M1: t = 9.09, *p* = 2.39 × 10^−8^; monkey M2: t = 4.38, *p* = 3.23 × 10^−4^). Anatomically defined parietal area PFG showed significant responses only in monkey M2 for observing actions in the action observation localizer (monkey M1: t = 2.06, *p* = 0.054; monkey M2: t = 3.15, *p* = 0.0053).

### 3.3. Cross-Modal fMRI Adaptation in Regions of Interest

To investigate potential cross-modal adaptation effects in these anatomical ROIs, we examined whether any ROI showed a significant reduction in response in congruent (action observation preceded by execution of the same action) compared to incongruent conditions (action observation preceded by execution of a different action). Thus, if mirror neuron regions were to show cross-modal fMRI adaptation effects, the difference between incongruent and congruent conditions should be significantly higher than zero. We plot this difference by subtracting the percentage signal change for the congruent condition from the incongruent condition (Figure 4). We found that there was no significant difference between action observation in congruent compared to incongruent conditions in parietal area AIP (monkey M1: Figure 4A, t = 0.42, *p* = 0.68; monkey M2: Figure 4B, t = −0.45, *p* = 0.65). Furthermore, the Bayes factor [22] in favor of the null hypothesis (BF_0_) for AIP was found to be 6.30 for monkey M1 and 6.43 for monkey M2, indicating that, given the data, for either monkey, it is ~6 times more probable that there is no cross-modal adaptation effect in AIP than that there is an effect. Similar results were also observed for area PFG (monkey M1: t = −0.28, *p* = 0.78, BF_0_ = 6.6; monkey M2: t = −0.18, *p* = 0.86, BF_0_ = 6.97), premotor F5 (monkey M1: t = −0.14, *p* = 0.89, BF_0_ = 6.79; monkey M2: t = −1.36, *p* = 0.18, BF_0_ = 2.96), area 44 (monkey M1: t = 1.45, *p* = 0.15, BF_0_ = 2.55; monkey M2: t = −0.19, *p* = 0.85, BF_0_ = 6.96), and prefrontal regions 45B (monkey M1: t = 0.95, *p* = 0.35, BF_0_ = 4.47; monkey M2: t = 0.61, *p* = 0.54, BF_0_ = 5.93), and 45A (monkey M1: t = 0.40, *p* = 0.69, BF_0_ = 6.35; monkey M2: t = 0.14, *p* = 0.89, BF_0_ = 7.01).

To ensure we did not miss any modest but significant adaptation effects in a smaller, functionally responsive portion of the anatomical ROIs, we also defined smaller ROIs by selecting only the subset of voxels from each anatomical ROI that were active during both the action execution and action observation localizers (see Methods). Even in these restricted ROIs, there were no significant differences between congruent compared to incongruent trials in parietal areas AIP (monkey M1: Figure 4C, t = −0.09, *p* = 0.93, BF_0_ = 6.83; monkey M2: Figure 4D, t = −0.23, *p* =0.82, BF_0_ = 6.9) and PFG (monkey M1: t = −1.59, *p* = 0.12, BF_0_ = 2.1; monkey M2: t = −0.48, *p* = 0.63, BF_0_ = 6.34), premotor F5 (monkey M1: t = 0.06, *p* = 0.95, BF_0_ = 6.84; monkey M2: t = −1.17, *p* = 0.25, BF_0_ = 3.7), area 44 (monkey M1: t = 0.87, *p* = 0.39, BF_0_ = 4.78; monkey M2: t = −0.34, *p* = 0.74, BF_0_ = 6.7), and prefrontal regions 45B (monkey M1: t = 1.14, *p* = 0.26, BF_0_ = 3.71; monkey M2: t = 0.62, *p* = 0.54, BF_0_ = 5.9), and 45A (monkey M1: t = 0.65, *p* = 0.52, BF_0_ = 5.6; monkey M2: t = 0.05, *p* = 0.96, BF_0_ = 7.07) in either monkey.

## 4. Discussion

We examined repetition suppression effects in the rhesus monkey brain in an experiment using a cross-modal fMRI adaptation paradigm. We specifically tested whether motor-to-visual cross-modal adaptation was present in parieto-frontal mirror neuron regions, given that this type of adaptation is routinely included in human fMRI mirror neuron/system adaptation studies. While uni-modal visual or motor adaptation effects in brain regions containing mirror neurons might be associated with distinct populations of neurons [5], cross-modal visual-to-motor adaptation effects can initiate motor priming, making detection and interpretation of these types of repetition suppression effects potentially difficult [3,8]. Therefore, cross-modal motor-to-visual adaptation has been suggested as the critical feature for inferring action specificity or common coding of observed and executed actions in the brain [3,5,8].

To date, only a handful of studies have combined fMRI with repetition suppression to examine the presence (or absence) of mirror neurons in the human brain. Using an experiment where subjects either performed or observed rock–paper–scissor gestures, Dinstein et al. [5] demonstrated uni-modal visual and motor adaptation in regions that are considered homologous to the macaque parietal and frontal mirror neuron regions. However, they failed to demonstrate either motor-to-visual or visual-to-motor cross-modal adaptation. Chong and co-authors [3] employed a stimulus set of 60 pantomimed hand gestures to investigate cross-modal fMRI adaptation. Subjects performed a series of two to five hand gestures and subsequently observed the same number of *same* or *different* hand gestures. The authors examined IFG, IPL, and STS using a ROI approach and only found cross-modal motor-to-visual adaptation effects in the right IPL. Lingnau et al. [8] conducted an fMRI adaptation study to investigate action adaptation, employing various meaningless hand gestures, and found within-modality and cross-modal visual-to-motor adaptation in parietal and premotor ROIs. However, they failed to find motor-to-visual adaptation. Two additional studies tested uni-modal and cross-modal fMRI adaptation, targeting primarily IFG [7,41]. Kilner et al. [7] demonstrated subtle bi-directional cross-modal adaptation only in posterior IFG (at *p* < 0.05, uncorr.), while Press et al. [41] observed similar effects only in a part of IFG bordering BA44 and BA6, but not in other parts (BA44, BA47, and BA6). 

A recent study tested cross-modal motor-to-visual adaptation only, using an object-directed grasp with a mimicked (non-object-directed) grasp [4]. They found that the bilateral medial frontal cortex, and in a confined part of left BA44/45, showed adaptation effects, slightly anterior and ventral, with respect to the IFG locus of Kilner et al. [7]. Finally, a recent fMRI human adaptation study [6] examined both uni- and cross-modal fMRI adaptation employing two actions: button presses with the index finger or wrist turns. These authors reported uni-modal adaptation effects only in a portion of ipsilateral SMA and contralateral MTG and only at a lenient threshold (*p* < 0.05, uncorrected at both peak voxel and cluster level). However, they found no such effects in the parietal and frontal regions commonly considered to be part of the core human mirror system (IPL and IFG). Moreover, they also failed to find cross-modal fMRI adaptation effects. In summary, the findings from these human fMRI adaptation studies, employing a variety of action-adaptation paradigms, suggest that it is no simple matter to detect robust and consistent cross-modal motor-to-visual adaptation effects in the proposed human homologs (IFG and IPL) of the parieto-premotor macaque monkey mirror neuron regions.

Currently, there are limited data on whether monkey motor or mirror neuron regions show repetition suppression during either action execution or observation tasks. One study has investigated uni-modal visual repetition suppression in monkey premotor F5 neurons, during the observation of videos of human grasping actions [15]. While most F5 neurons did not vary their spiking activity between the first and second presentation of the video, the neurons that did show a variation showed an increase in firing, rather than a decrease as expected if the response was to be adapted. At the population level, there was little, if any, indication of a response decrease for the second presentation of the video. However, analysis of the local field potentials was suggestive of some reductions occurring at multiple, short time intervals during the second presentation of the video. A second study [16] also examined uni-modal visual adaption in F5 neurons and showed modest changes in firing rates and beta frequencies of LFPs for monkey F5 mirror neurons during action observation, but only with longer adaptation sequences where the visual stimulus (live actor) was repeated over 7–10 trials. Thus far, motor-to-motor or cross-modal (motor-to-visual or vice versa) repetition suppression at the single-cell or population level in mirror neuron (or motor) regions has not been investigated (or at least not described in detail) with monkey electrophysiology. 

Recently, another study from our lab examined if monkey parietal and premotor mirror neuron regions show either uni-modal or cross-modal fMRI adaptation [2]. In that study, two monkeys performed either a grasp/lift or a touch/press hand action, directed to the same object, or observed videos of a conspecific performing these two actions. While visual-to-visual adaptation was detected in early visual cortices, there was no evidence for uni- or cross-modal adaptation effects in parieto-frontal mirror neuron regions. In our current study, we employed a longer adaptation period, which has been suggested as being critical for observing subtle adaptation effects at the neural level in premotor F5 [16]. Furthermore, actions and action goals employed in the current study (grasp ball vs. touch table) were highly distinct. Most importantly, using the same action execution motor tasks and observation videos that we used in the current fMRI adaptation study, a previous monkey fMRI study employing multivariate pattern analysis did find evidence for cross-modal decoding in several monkey brain regions that house mirror neurons [1]. Thus, any absence of cross-modal fMRI adaptation effects that we might find could not be attributed merely to the ineffectiveness of the tasks in driving responses in mirror neuron regions. Finally, since cross-modal motor to visual fMRI adaptation is suggested as the most crucial test for demonstrating the presence of mirror neurons [3,5,8], we only tested for such effects in the current study. In doing so, we ensured that we did not miss out on any weak adaptation effects due to a lack of power. However, despite using a different adaptation paradigm and motor tasks from Cui and Nelissen [2], we also failed to find cross-modal fMRI adaptation effects in either monkey in our current study, even at extremely liberal thresholds. Thus, while further experiments using different tasks and scanning paradigms are needed to make definitive claims, these findings suggest that MVPA might be a more suitable technique for investigating mirror neuron responses using fMRI [6,42]. 

While there are several reasons that make interpretation of the presence or absence of uni-modal action adaptation effects difficult, the detection of cross-modal motor to visual adaptation may additionally pose certain challenges. For instance, unlike action observation, action execution generally elicits much higher fMRI responses, particularly in (pre)motor regions. Given the relatively prolonged nature of the fMRI signal, it would be challenging to distinguish subtle modulations in the action observation condition from previously induced execution-related responses in motor areas [2]. In addition, it has been suggested that even if adaptation were to occur in visuo-motor mirror neurons at the synaptic level, it might not be easy to discern cross-modal adaption effects [5,43]. Specifically, though visual information possibly reaches premotor F5 neurons through an STS–parietal–premotor pathway during action observation [44], it is possible that during action execution, F5 neurons might receive inputs from frontal or other cortical and subcortical motor regions. Therefore, it might be improbable that mirror neurons receive shared input at the synaptic level because of action observation and execution tasks [45].

A shortcoming of the proposal of cross-modal (motor-to-visual) fMRI adaptation being a key characteristic of mirror neurons is that it rests on several theoretical assumptions that have not yet been tested: (1) Does motor-to-visual adaptation indeed occur in visuo-motor neurons in the same way that neural adaptation occurs in sensory neurons when these are repeatedly stimulated using sensory stimuli? (2) Do neurons that respond to action execution and observation show this type of cross-modal motor to visual adaptation and at which time scale? (3) If these adaptation signals are present at the single-cell level, is fMRI sensitive enough to register these adaptation signals? Single-cell adaptation studies in monkeys will be crucial to answer some of these questions. 

In conclusion, our study confirms that monkey mirror neuron activity does not readily translate into detectable cross-modal motor-to-visual fMRI repetition suppression effects. These findings highlight the need for caution when employing cross-modal fMRI adaptation to draw conclusions about the existence of mirror neurons in the primate brain.

## Figures and Tables

**Table 1:** The number of voxels in the anatomical and intersected ROIs for monkey M1 and monkey M2.

	Monkey M1		Monkey M2	
	Anatomical	Intersected	Anatomical	Intersected
AIP	113	13	102	28
PFG	108	17	90	34
F5	409	176	415	79
44	49	27	50	19
45b	107	80	98	60
45a	79	49	63	47

## Data Availability

Datasets will be made available on reasonable request.
